# A Retrospective Study of Preferable Alternative Route to Right Internal Jugular Vein for Placing Tunneled Dialysis Catheters: Right External Jugular Vein versus Left Internal Jugular Vein

**DOI:** 10.1371/journal.pone.0146411

**Published:** 2016-01-11

**Authors:** Pei Wang, Yufei Wang, Yingjin Qiao, Sijie Zhou, Xianhui Liang, Zhangsuo Liu

**Affiliations:** Department of Nephrology, Blood purification center, The First Affiliated Hospital of Zhengzhou University, Zhengzhou, China; University of Utah School of Medicine, UNITED STATES

## Abstract

**Background:**

Right internal jugular vein (IJV) is a preferred access route for tunneled (cuffed) dialysis catheters (TDCs), and both right external jugular vein (EJV) and left IJV are alternative routes for patients in case the right IJV isn’t available for TDC placement. This retrospective study aimed to determine if a disparity exists between the two alternative routes in hemodialysis patients in terms of outcomes of TDCs.

**Methods:**

49 hemodialysis patients who required TDCs through right EJV (n = 21) or left IJV (n = 28) as long-term vascular access were included in this study. The primary end point was cumulative catheter patency. Secondary end points include primary catheter patency, proportion of patients that never required urokinase and incidence of catheter-related bloodstream infections (CRBSI).

**Results:**

A total of 20,870 catheter-days were evaluated and the median was 384 (interquartile range, 262–605) catheter-days. Fewer catheters were removed in the right EJV group than in the left IJV group (*P* = 0.007). Mean cumulative catheter patency was higher in the right EJV group compared with the left IJV group (*P* = 0.031). There was no significant difference between the two groups in the incidence of CRBSI, primary catheter patency or proportion of patients that never required urokinase use. Total indwell time of antecedent catheters was identified as an independent risk factor for cumulative catheter patency by Cox regression hazards test with an HR of 2.212 (95% CI, 1.363–3.588; *p* = 0.001).

**Conclusions:**

Right EJV might be superior to left IJV as an alternative insertion route for TDC placement in hemodialysis patients whose right IJVs are unavailable.

## Introduction

Vascular access is a major issue in chronic hemodialysis patients[1]. Arteriovenous fistula (AVF) is widely recognized as preferred access for most hemodialysis patients because it provides the best outcomes compared with an arteriovenous graft (AVG) or tunneled cuffed dialysis catheter (TDC)[2]. Catheter access has been used in 10% hemodialysis patients in China[3, 4] and in 15% hemodialysis patients in the US according to the recent Dialysis Outcomes and Practice Patterns Study (DOPPS) Practice Monitor (DPM) data, despite a great effort to reduce its use.

It has been recommended by the NKF-KDOQI (National Kidney Foundation–Kidney Disease Outcomes Quality Initiative) clinical practice guidelines that right internal jugular vein (IJV) is a preferred route for hemodialysis catheters[2], for its ease in identification, big size and unhindered straight passage to the right atrium[5]. When the right IJV is not available for TDC placement, the second access route remains variable. Although the left IJV and both subclavian vein (SCV) have been used for secondary access by most clinicians, several studies suggest that both the SCVs and left IJV should be avoided because of a high incidence of procedural complications as well as central stenosis and thrombosis[6, 7]. The right external jugular vein (EJV) as access for hemodialysis as well as non-dialysis central vein catheter placement has been reported[8–12]. Its relatively straightforward course, superficial location and similar blood flow as IJV are main advantages for its use[8].

In this retrospective study, the patency and infection rate of TDCs through right EJV versus left IJV were compared in hemodialysis patients to determine whether difference exists between right EJV and left IJV route for TDC long term placement.

## Materials and Methods

### Study population

**Fig 1:**
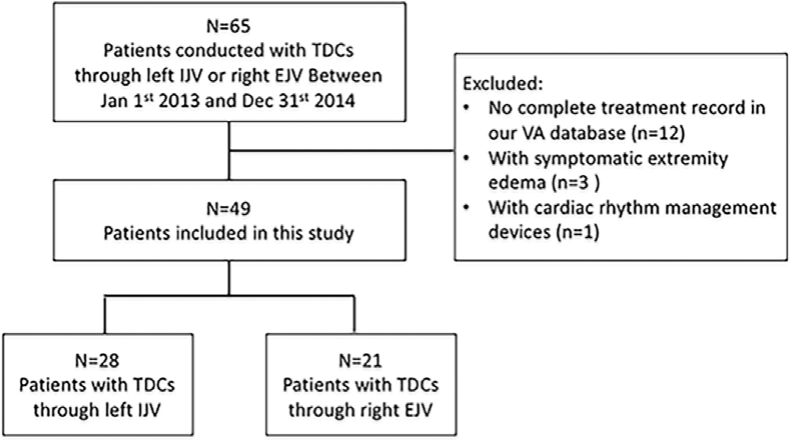
Study diagram. Abbreviations: IJV, internal jugular vein; EJV, external jugular vein; VA, vascular access; TDCs, tunneled cuffed dialysis catheters.

We carried out a retrospective case control study of patients with TDCs through right EJV (n = 21) or left IJV (n = 28) placed from January 1, 2013 to December 31, 2014 in the First Affiliated Hospital of Zhengzhou University. The study patients consisted of adult (aged ≥ 18 years) chronic hemodialysis patients who had TDCs through right EJV or left IJV as long-term vascular access. We excluded patients who had: (1) no complete treatment record in our vascular access database, (2) with symptomatic bilateral or unilateral extremity edema, (3) with cardiac rhythm management devices, peripherally inserted central catheters, or other central venous catheters or ports (Fig 1). This study was approved by the institutional review board of the First Affiliated Hospital of Zhengzhou University with no written consent because patients’ records/information was anonymized and de-identified prior to analysis. All clinical investigations were conducted according to the 2008 Declaration of Helsinki and good clinical practice guidelines.

### Standard of procedures for intravenous catheterization and care of catheters

The baseline data were collected retrospectively. Before insertion procedure, IJV, EJV, brachiocephalic vein (BCV), subclavian vein (SBV) and superior vena cava (SVC) were evaluated by Doppler-ultrasonography (DUS). Thrombosis and stenosis were recoded, and most patients had a high-grade right IJV stenosis, defined as >50% stenosis of the right IJV cross-section[13]. Chest and extremity were carefully examined for dilated collateral veins and obvious edema. All catheter placement attempts were guided by DUS, and more than half (29 cases) were aided by percutaneous angiography (PTA). Symmetric tip catheters (Palindrome, Covidien), which have an outer diameter of 14.5F and a single cuff, were used for all left IJV catheterization and for 7 right EJV catheterization. For the other 14 right EJV catheterization, split tip catheters (Cannon II Plus, Arrow), which have an outer diameter of 15F and a single cuff, were used. TDC was inserted through left IJV with Seldinger technique, while through right EJV with Seldinger (14 cases) or surgical technique (7 cases) according to the patients’ condition (S1 Fig). Heparin (2500-5000IU/ml) was used as the catheter lock solution in all catheters. Antiplatelet was recommended for patients with no evidence of hemorrhage risk. Low molecular weight heparin was the anticoagulation agent for all hemodialysis sessions unless the risk of hemorrhage emerged.

### Catheter dysfunction and removal

Catheter dysfunction was defined as one or more of the following conditions: 1) failure to achieve a pump blood flow rate < 250mL/min for more than 30 minutes, with arterial pressure less than −250 mm Hg or venous pressure > 250 mm Hg, 2) inability to initiate dialysis owing to inadequate blood flow, after attempts to restore patency have been attempted, 3) requirement of three times or more of urokinase infusion prior to any session in any month[14, 15]. In case of catheter dysfunction, urokinase was used to rescue the catheter and this was recorded in patients’ charts and databases. When urokinase was applied thrice within a time frame of two weeks and the catheter was still dysfunctional, catheter removal and exchange was considered.

### Catheter related bloodstream infections (CRBSI) and catheter removal

Catheter-related bloodstream infection (CRBSI) was defined as the presence of systemic signs and symptoms of infection (fever or chills) in combination with positive blood cultures without clinical evidence for an alternative source of infection[16]. Blood cultures were drawn from both the catheters and a peripheral vein in case of patients with clinical signs of suspected infection (presenting with systemic inflammatory symptoms such as fever with or without chills or elevated white blood cell count)[16]. CRBSI episodes and antibiotics administration was recorded in patients’ charts and databases. The database did not include information about exit site or tunnel infection. Catheter removal was considered in case of sustained CRBSI that persisted for more than 48 hours after initiation of antibiotics, or if the patient had positive surveillance blood cultures one week after completing the antibiotic course.

### Outcomes and Definitions

Primary end point was the cumulative catheter patency, which was the time period commencing from catheter insertion to the time of removal for any reason[14]. Secondary end points include the primary catheter patency, urokinase use and incidence of catheter-related bloodstream infections (CRBSI). The primary catheter patency was the time period commencing from catheter insertion to the time of first intervention for the same catheter[14]. Incidence of CRBSI was defined as the number of events per 1,000 catheter-days.

### Statistical analysis

Using SPSS 22.0 software, baseline patient characteristics were compared using independent t test or Wilcoxon rank sum test for continuous variables and chi-square test for categorical variables. Primary and secondary catheter patency was estimated by the Kaplan-Meier method. Log rank is used for the comparison of outcome proportions between groups. Multivariate analysis was employed using the Cox proportional hazards model to examine the independent factors of catheter events. A p-value of <0.05 was considered statistically significant.

## Results

### Study Population

A total of 20,870 (9121 in right EJV group and 11749 in left IJV group) catheter-days were evaluated during the period of this study (from January 1, 2013, to March 31, 2015). Median follow-up time was 384 (interquartile range [IQR], 262–605) days. All patients had at least one prior catheter placement in right IJV, and 19(38.8%) had three or more times of prior catheterization in right IJV. The median of indwell time of antecedent catheters was 12 (IQR, 8–16) months. Baseline characteristics of the patients between the two groups were comparable with regard to age, gender, body weight, dialysis vintage, proportion of diabetes, reasons for catheter replacement, the times of prior right IJV insertion times, and indwell time of antecedent catheters (Table 1, S1 Table).

**Table 1:** Baseline demographic and clinical variables of the study patients.

Variables	All	Left IJV	Right EJV	P-value ^a^
	N = 49	N = 28	N = 21	
Follow-up (days)	384(262–605)	379(258–644)	444(269–601)	0.800
Age (years)	66(58.5–73)	66(58.3–70.3)	65(60–73)	0.820
Male sex	26(53.1)	15(53.6)	11(52.4)	0.777
Body weight (Kg)	58(51–66)	60(52–67)	58(49–67)	0.782
Dialysis vintage (months)	23(15–36.5)	24.5(15–41.8)	25(19–31)	0.237
Diabetes	22(44.9)	12(42.9)	10(47.6)	0.478
Reason for catheter replacement				
Infection	15(30.6)	8(28.6)	7(33.3)	0.357
Dysfunction	18(36.7)	11(39.3)	7(33.3)	
AVF dysfunction	12(24.5)	8(28.6)	4(19.0)	
Indwell time of antecedent catheters (months)	11(8–16)	9(8–16)	12(9–16)	0.542
Right IJV catheterization ≥3	19(38.8)	9(32.1)	10(47.6)	0.021
High-grade right IJV stenosis ^b^	44(89.8)	25(89.3)	19(90.5)	0.799
Thrombosis in BCV/SCV ^b^	10(20.4)	7(25)	3(14.3)	0.050
Dilated collateral veins ^c^	8(16.3)	5(17.9)	3(14.3)	0.440
Aided by PTA	29(59.2)	15(53.6)	14(66.7)	0.060
TDCs				
Symmetric tip(Palindrome)	35(71.4)	28(100)	7(33.3)	NA
Split tip (Cannon II Plus)	14(28.6)	0(0)	14(66.7)	
Vein cut-down	7(14.3)	0(0)	7(33.3)	NA
Antiplatelet	38(77.6)	21(75.0)	17(81.0)	0.306

Note: Values for categorical variables are given as number (percentage); values for continuous variables are given as median [interquartile range].

Abbreviations: IJV, internal jugular vein; EJV, external jugular vein; AVF, arteriovenous fistula; TDC, tunneled (cuffed) dialysis catheters; BCV, brachiocephalic vein; SCV, subclavian vein; PTA, percutaneous angiography.

a According to independent t test, Wilcoxon test or chi-square test.

b Vessels were examined by Doppler-ultrasonography prior to procedure.

c Seen on right chest or extremity clearly, excluded other reasons.

### Catheter removal

Totally, 8 catheters (38.1%) were removed in right EJV group, of which 5 (23.8%) were removed due to catheter dysfunction. In stark contrast, 16 catheters (57.1%) were removed in left IJV group (HR, 0.677; 95% CI, 0.505–0.908, *P* = 0.007), of which 12 patients (42.9%) were removed due to catheter dysfunction (HR, 0.627; 95% CI, 0.441–0.892, *P* = 0.004, Table 2).

**Table 2:** Outcomes of study.

	All	Left IJV	Right EJV	P-value^a^
	N = 49	N = 28	N = 21	
Catheter removal				
All	24(49.0)	16(57.1)	8(38.1)	0.007
CRBSI	7(15.3)	4(14.3)	3(14.3)	1.000
Dysfunction	17(34.7)	12(42.9)	5(23.8)	0.004
Cumulative catheter patency (days)		261.36±20.81	382.04±33.55	0.031
Primary catheter patency (days)		200.20±23.31	251.75±19.66	0.211
Never required urokinase	15(30.6)	8(28.6)	7(33.3)	0.541
CRBSI patients	10(20.4)	5(17.9)	5(23.8)	0.298
CRBSI rate	0.86	0.83	0.89	0.860

Note: Values for categorical variables are given as number (percentage); values for continuous variables are given as mean ± standard deviation. Catheter-related bloodstream infection (CRBSI) rate is given as number/1,000 catheter-days.

a According to independent t test, Wilcoxon test or chi-square test.

### Cumulative catheter patency

**Fig 2:**
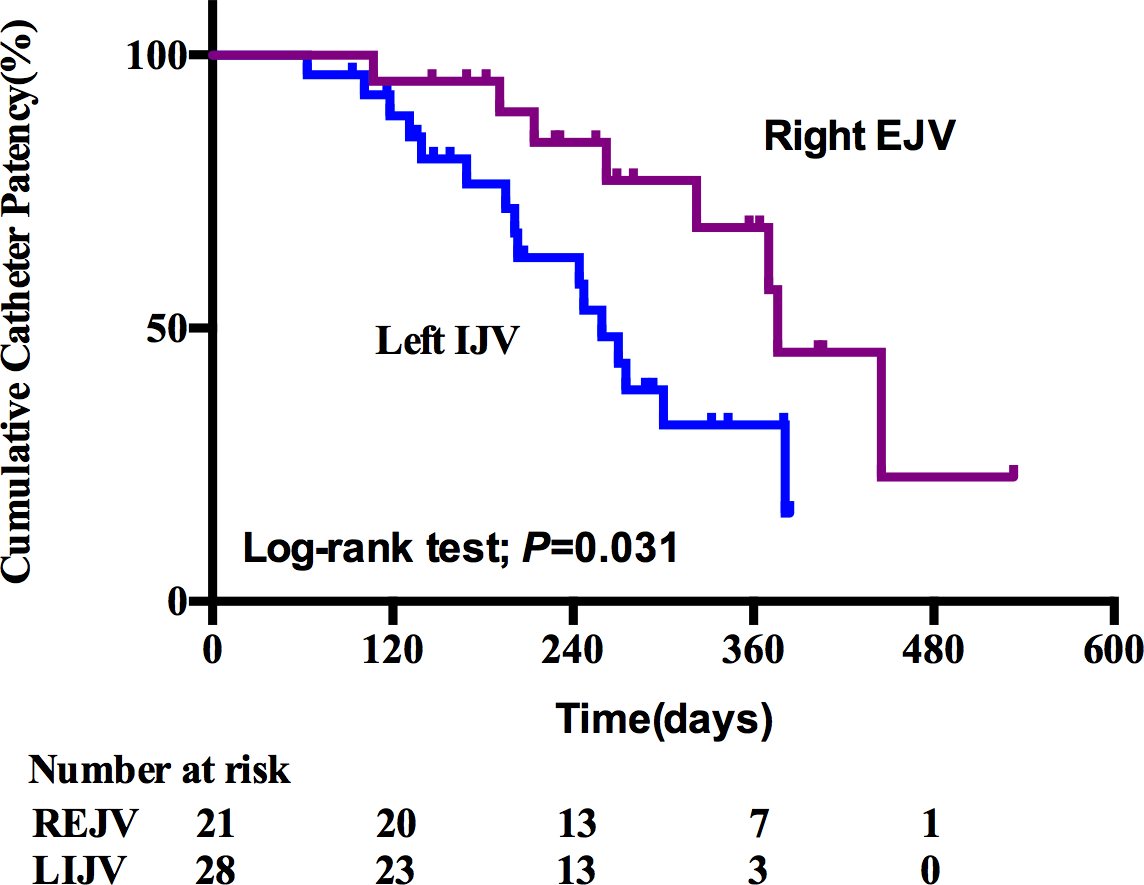
Kaplan-Meier curve of cumulative catheter patency. Patients with TDCs through left IJV (Blue, n = 28) were compared with those through right EJV (purple, n = 21). TDCs, tunneled cuffed dialysis catheters; IJV, internal jugular vein; EJV, external jugular vein.

Mean cumulative catheter patency was significantly higher in right EJV group compared with that in left IJV group (382.04±33.55 vs. 261.36±20.81 days, *P* = 0.031), Table 2, Fig 2). Catheter survival at 240 days was 61.9% in right EJV group vs. 46.4% in left IJV group (HR, 1.390; 95% CI 1.037–1.863; *P* = 0.023), and at 360 days was 33.3% vs. 10.7% (HR, 1.746; 95% CI 1.362–2.239; *P* = 0.000), respectively. Indwell time of antecedent catheters was identified as an independent risk factor for cumulative catheter patency by Cox regression hazards test with a HR of 2.212 (95% CI, 1.363–3.588; *P* = 0.001, Table 3).

**Table 3:** Association of cumulative catheter patency with clinical parameters.

Variable	Hazard Ratio(95%CI)	P-value
Age, >65 vs ≤65	0.676(0.205–2.232)	0.520
Sex, female vs male	0.347(0.088–1.367)	0.130
Diabetes	1.282(0.336–4.889)	0.716
Dialysis Vintage, >24months vs ≤24months	0.477(0.166–1.376)	0.171
Indwell time of antecedent Catheterization, increase every 6months vs ≤6months	2.212(1.363–3.588)	0.001
Three times or more of antecedent catheterization, >2 vs ≤2	0.949(0.293–3.068)	0.930
Thrombosis in BCV/SCV	1.787(0.414–7.711)	0.436
Intervention of PTA	0.452(0.114–1.796)	0.259
Antiplatelet	1.363(0.327–5.682)	0.670

Note: Cox proportional hazards model performed for independent factors of cumulative catheter patency.

Abbreviations: BCV, brachiocephalic vein; SCV, subclavian vein; PTA, percutaneous angiography, HR, hazard ratio; CI, confidence interval.

### Primary catheter patency

**Fig 3:**
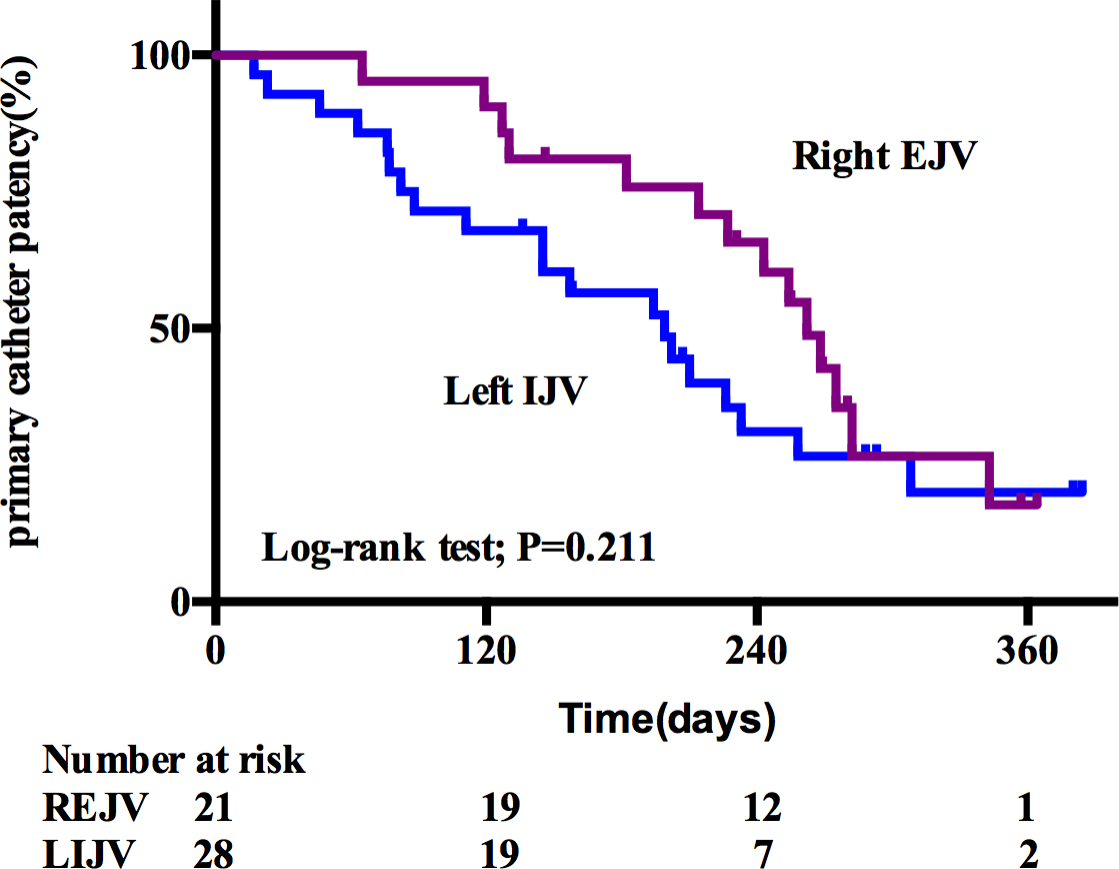
Kaplan-Meier curve of primary catheter patency. Patients with TDCs through left IJV (Blue, n = 28) were compared with those through right EJV (purple, n = 21). TDCs, tunneled cuffed dialysis catheters; IJV, internal jugular vein; EJV, external jugular vein.

Mean primary catheter patency was 251.75±19.66 days for the TDCs through right EJV and 200.20±23.31 days for TDCs through left IJV (*P* = 0.211). The proportion of catheters that never required urokinase at 240d was 57.1% in right EJV group vs. 25.0% in left IJV group (HR, 3.977; 95% CI 2.180–7.256; *P* = 0.000) respectively (Table 2, Fig 3). Total indwell time of antecedent catheters was identified as an independent risk factor for primary catheter patency by Cox regression hazards test with a HR of 2.100 (95% CI, 1.412–3.122; *P* = 0.000) (Table 4).

**Table 4:** Association of primary catheter patency with clinical parameters.

Variable	Hazard Ratio(95%CI)	P-value
Age, >65 vs ≤65	1.150(0.449–2.943)	0.771
Sex, female vs male	0.480(0.191–1.202)	0.117
Diabetes	0.688(0.278–1.704)	0.419
Dialysis Vintage, >24months vs ≤24months	0.586(0.247–1.390)	0.225
Indwell time of antecedent catheterization, >12months vs ≤12months	2.100(1.412–3.122)	0.000
Three times or more of antecedent catheterization	0.587(0.213–1.619)	0.303
Thrombosis in BCV/SCV	0.720(0.224–2.312)	0.581
Intervention of PTA	0.567(0.197–1.633)	0.293
Antiplatelet	1.715(0.586–5.017)	0.325

Note: Cox proportional hazards model performed for independent factors of primary catheter patency.

Abbreviations: BC, brachiocephalic vein; SCV, subclavian vein; PTA, percutaneous angiography, HR, hazard ratio; CI, confidence interval.

### Urokinase use

The proportion of patients that never required urokinase use was not significantly different between the two groups, 7 cases (33.3%) in right EJV group vs 8 cases (28.6%) in left IJV group (*P* = 0.056, Table 2).

### Catheter related blood stream infection (CRBSI)

The incidence of CRBSI had no significant difference between the two groups (0.83 in left IJV group vs 0.89 in left IJV group per 1000 catheter-day; *P* = 0.860, Table 2).

## Discussion

Our study demonstrates that TDCs through right EJV was associated with a higher cumulative catheter patency and a less risk of catheter removal than through left IJV. There was no significant difference between the two groups in the incidence of CRBSI, primary catheter patency and proportion of patients that never required urokinase use.

In spite of the fact that TDC is discouraged in the NKF-KDOQI guidelines, catheter dependent hemodialysis is not uncommon in clinical practice. According to a recent cross-section study of a total number of 10646 prevalent hemodialysis patients in Henan (a province in central China), tunneled catheters and non-tunneled catheters were used as vascular access for hemodialysis respectively in 1865(17.5%) and 565 (5.3%) patients. However, due to long time catheter indwell or repeated catheterization, IJV occlusion or stenosis develops and results in failure of IJV attempts or catheter dysfunction[17, 18]. In fact, most of TDCs (583/637, 91.5%) were inserted to patients with a history of at least one time of right IJV placement in our center from January 1, 2013 to December 31, 2014. In a cohort of 143 TDCs through right IJV with an average indwell time of 10.3 months, 37 (25.8%) had thrombosis, 23 (16.1%) had occluded IJV[19]. Although longer catheter indwell time has been implicated in central vein stenosis, short-term non-tunneled dialysis catheters also have been associated with this complication. Indeed, Naroienejad et al reported an incidence of vein stenosis of 18% in patients with non-tunneled double-lumen dialysis catheters[20]. So there are urgent needs for an optimal alternative route for placement of TDCs in patients whose right IJV are no longer usable.

Left IJV and right EJV are among the strongest candidate alternatives to right IJV due to lower risk of thrombosis and infection complications and are technically more accessible compared to others, e.g., femoral, subclavian, translumbar, and transhepatic veins[2, 21]. A report from Vats et al compared blood flow outcomes at 90 days of catheters in right EJV (n = 16), left IJV (n = 13) and right IJV (n = 17), and found there were no differences among the three groups, indicating TDC through both right EJV and left IJV may be equally long-term alternative vascular accesses for dialysis patients[9]. Another study by Skandalous et al reported their experience with external jugular catheters in 168 hemodialysis patients. Unfortunately, the indication for use of EJV and long-term outcomes were not reported in their study[10]. Our data also demonstrated a similar outcome in the incidence of CRBSI, primary catheter patency and proportion of patients that never required urokinase between TDCs through right EJV and those through left IJV.

A better cumulative catheter patency was associated with TDC through right EJV in our study. And the proportion of catheter removal caused by catheter dysfunction was markedly less in right EJV group than in left IJV group. Higher incidence of thrombosis and catheter dysfunction was associated with TDC through left IJV compared with right IJV[2, 22, 23]. The underlying reason for this finding is unknown, but TDC through left IJV encounters at least three incurvation of opposite convexity to reach the right atrium[6]. In contrast, right EJV, similar to right IJV provides a much smoother route to right atrium. Thus, it’s conceivable that TDC via right EJV might offer a better outcome, such as higher cumulative catheter patency and lower incidence of catheter removal, as shown in the present study and by other reports[9]. Another inferiority of a TDC through left IJV is that it may impact the maturation and survival of AVF, which is typically placed in the non-dominant (usually left) arm[23, 24]. Occlusion of the BCV or SCV is a potential risk of TDC through right EJV, which shares right BCV with right IJV. It’s important to perform DUS examination and physical examination prior to procedure in order to improve the success of placement[2].

Our study has some limitations due to the retrospective nature. First, participants were not allocated randomly to the two groups. Optimal regimen was administrated to patients according to the DUS examination and physical examination prior to procedure. There was relative higher incidence of thrombosis in right BCV/SCV in left IJV group than in right EJV group, although this difference had no statistical significance. The effects of thrombosis in right BCV/SCV on the TDCs through left IJV were not examined. However, in our study, multivariate Cox regression hazards test revealed that thrombosis in right BCV/SCV is less likely to be an independent risk factor for cumulative catheter patency (Table 3). Second, to identify a catheter dysfunction, pump blood flow rate was defined as less 250 mL/min in this study, not 300 mL/min as recommended by NKF-DOQI. Nevertheless, Moist et al reported that a blood flow rate of <300 mL/min may deliver adequate dialysis in a population with an average body weight of 71 Kg[25]. Patients in our study had a much smaller body weight of 58 Kg and might need a lesser pump blood flow rate to achieve adequate dialysis in our population. For very few patients who failed to achieve an adequate dialysis even with a pump blood flow rate more than 250 ml/min, a catheter dysfunction was suspected and validated. Finally, this was a single-center study, thus the findings may not be generalized to other dialysis center and merit further validation by large scale multicenter prospective trails.

In conclusion, TDC through right EJV provided a better outcome than through left IJV, with a higher cumulative patency and a less risk of catheter removal in hemodialysis patients whose right IJV was unavailable due to repeated or long-time use. Our study suggests that right EJV might be superior to left IJV in serving as a preferable alternative vascular access route for TDC placement.

## Supporting Information

S1 Fig Standard of procedures for intravenous catheterization and care of catheters. (TIF)

S1 Table Detailed clinical data of the individual study subjects. (PDF)
