# Supplementary figures and images for: A Retrospective Study of Preferable Alternative Route to Right Internal Jugular Vein for Placing Tunneled Dialysis Catheters: Right External Jugular Vein versus Left Internal Jugular Vein
Source: PLoS One. 2016 Jan 11;11(1):e0146411. doi: 10.1371/journal.pone.0146411 (PMC4709068; doi:10.1371/journal.pone.0146411)

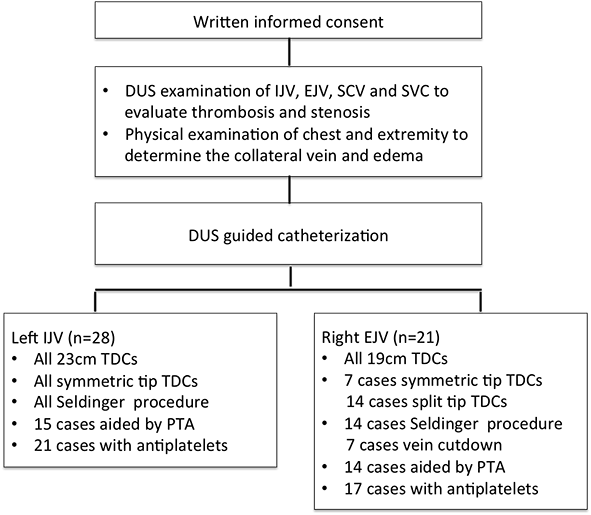

Supplement: S1 Fig — (TIF) [file pone.0146411.s001.tif]
