# Supplementary material for: A Retrospective Study of Preferable Alternative Route to Right Internal Jugular Vein for Placing Tunneled Dialysis Catheters: Right External Jugular Vein versus Left Internal Jugular Vein
Source: PLoS One. 2016 Jan 11;11(1):e0146411. doi: 10.1371/journal.pone.0146411 (PMC4709068; doi:10.1371/journal.pone.0146411)
Supplement: S1 Table — (PDF) [file pone.0146411.s002.pdf]

patients	group	age	gender	bw	DM	vintage
50	1	51	2	54	1	21
49	1	58	1	69	2	51
48	1	66	1	79	2	49
47	1	72	1	65	2	11
46	1	66	1	70	1	35
45	1	68	2	49	1	24
44	1	75	2	52	2	53
43	2	63	1	72	2	34
42	1	68	2	43	2	21
41	1	57	1	69	1	22
39	2	66	1	58	2	31
38	1	58	2	65	1	25
37	2	48	2	78	1	20
36	2	73	2	48	2	19
35	1	79	2	45	2	31
34	1	71	1	66	2	18
33	2	67	1	56	1	51
32	1	59	2	57	1	51
31	2	62	2	51	1	10
30	1	60	1	68	2	41
29	2	62	2	44	1	10
28	2	49	1	71	1	9
27	1	72	1	57	2	42
26	1	73	2	42	2	48
25	1	41	2	53	1	23
24	2	76	2	58	2	18
23	2	82	1	64	2	17
22	1	80	2	55	1	5
21	2	60	1	52	2	16
20	2	51	2	47	1	41
19	1	63	1	59	2	15
18	2	68	2	48	1	31
17	2	73	1	60	2	16
16	1	66	2	48	1	36
15	1	59	1	61	2	11

14	1	75	1	66	2	39
13	2	57	1	82	2	12
12	1	58	2	66	1	17
11	2	70	2	41	1	6
10	2	61	2	49	1	21
9	1	72	1	67	1	6
8	2	77	1	58	2	31
7	2	65	1	59	2	15
6	1	62	1	63	2	26
5	2	49	2	65	1	29
4	1	76	1	56	2	15
3	2	83	1	67	2	37
2	1	74	2	51	2	9
1	1	55	1	65	1	37

reason	VAtype	dwell interval	times of cath	cattype	tinbcv
1	3	9	2	1	1
4	1	9	2	1	2
2	2	15	3	1	2
2	2	8	2	1	1
4	1+5	6	2	1	1
4	1+5	8	2	1	1
1	2	12	2	1	1
4	1+5	6	1	1	2
1	3	21	5	1	1
2	4	16	4	1	1
2	4	13	2	2	1
1	2	9	2	1	1
2	2	29	3	2	1
2	3	9	3	2	1
4	1	6	2	1	1
2	4	18	2	1	2
4	1	2	2	1	1
4	1	2	1	1	1
1	4	10	2	2	1
2	2	8	2	1	2
1	2	10	2	1	1
1	4	9	2	1	1
2	2	24	4	1	1
2	2	12	4	1	2
1	3	13	4	1	1
1	4	13	3	2	1
3	3	17	2	2	1
4	1	5	2	1	1
1	2	16	3	1	1
4	1	3	1	2	1
1	2	15	2	1	1
2	3	21	4	2	2
1	2	10	3	1	1
4	1	6	2	1	1
1	2	11	2	1	1

2	2	16	4	1	2
2	2	12	2	2	2
2	2	31	3	1	1
3	3	6	2	1	1
4	1	6	2	2	1
3	3	22	2	1	1
2	2	15	3	2	1
2	2	25	3	2	1
4	1	5	1	1	1
1	3	13	3	2	1
2	3	9	3	1	1
3	2	37	5	2	1
1	2	9	2	1	1
2	5	21	1	1	2

colvein	PTA	veincut	antipla	followup	death	death interval
2	2	1	2	158	2	
1	2	1	2	288	2	
2	2	1	2	162	2	
1	1	1	2	116	2	
1	1	1	1	343	2	
1	1	1	1	147	2	
1	1	1	2	248	2	
1	2	1	2	280	2	
1	2	1	2	808	1	93
1	1	1	2	400	2	
1	1	1	2	146	2	
1	2	1	2	136	2	
1	2	1	2	169	2	
1	1	1	2	357	2	
1	1	1	1	456	2	
1	2	1	2	353	2	
1	1	1	2	228	2	
1	1	1	2	207	2	
1	2	2	2	269	2	
1	2	1	2	587	2	
2	2	2	2	406	2	
1	2	2	2	231	2	
2	1	1	2	609	1	169
1	2	1	2	623	1	244
1	2	1	2	651	1	300
1	1	1	2	255	2	
1	2	1	1	641	2	
1	1	1	1	293	2	
1	2	2	2	511	2	
1	1	1	2	533	2	
1	2	1	2	678	1	201
2	2	2	2	560	1	364
1	2	2	2	601	2	
1	2	1	2	332	2	
1	2	1	1	721	2	

2	2	1	2	736	1	381
1	2	1	2	444	1	191
1	1	1	2	377	2	
1	1	1	1	691	1	403
1	1	2	2	646	2	
1	2	1	2	755	1	101
1	2	1	1	355	1	214
1	2	1	2	514	2	
1	1	1	2	384	2	
2	2	1	2	478	2	
1	1	1	1	426	1	322
1	2	1	1	806	1	182
1	1	1	1	380	2	
2	2	1	2	375	2	

urokinase	urokinase interval	CRBSI	CRBSI interval	VA change
2	158	0	158	2
2	288	0	288	2
1	63	0	139	1
1	46	0	116	2
2	343	0	343	2
1	76	0	147	2
1	145	1	188	1
2	280	0	280	2
1	88	1	30	2
1	77	1	84	1
2	146	0	146	2
2	136	0	136	2
1	127	0	169	2
2	357	0	357	2
1	99	0	259	1
1	226	0	247	1
1	214	0	228	2
2	207	0	207	2
2	269	0	269	2
1	82	0	131	1
1	254	1	27	2
2	231	1	181	2
1	111	0	169	1
1	202	0	244	1
1	210	1	295	1
2	255	0	255	2
1	227	0	262	1
2	293	0	293	2
1	275	1	351	1
1	268	0	533	2
1	45	0	201	1
2	364	0	364	2
1	343	0	376	1
1	233	0	332	2
1	258	0	275	1

1	308	1	334	1
1	130	0	191	1
1	17	0	63	1
1	243	0	403	2
1	262	0	445	1
1	23	0	101	1
1	182	0	214	1
1	65	1	79	1
2	384	0	384	2
1	282	1	319	1
1	194	0	270	1
1	119	0	182	2
2	380	0	380	2
1	157	0	203	1

interval for VA change	reason for VA change	intervaltoep
		158
		288
139	2	139
		116
		343
		147
195	1	195
		280
		93
118	1	118
		146
		136
		169
		357
259	2	259
247	2	247
		228
		207
		269
131	2	131
		406
		231
169	2	169
244	2	244
300	1	300
		255
262	2	262
		293
370	1	370
		533
201	2	201
		364
376	2	376
		332
275	2	275

381	1	381
191	2	191
63	2	63
		403
	2	445
101	2	101
214	2	214
107	1	107
		384
322	1	322
270	2	270
		182
		380
203	2	203
